# Impact of childhood 13-valent pneumococcal conjugate vaccine introduction on adult pneumonia hospitalisations in Mongolia: a time series analysis

**DOI:** 10.1016/j.lanwpc.2023.100983

**Published:** 2023-12-11

**Authors:** Kirsten Fagerli, Munkhchuluun Ulziibayar, Bujinlkham Suuri, Dashtseren Luvsantseren, Dorj Narangerel, Purevsuren Batsaikhan, Bilegtsaikhan Tsolmon, John de Campo, Margaret de Campo, Eileen M. Dunne, Kristen E. Allen, Anneke C. Grobler, Cattram D. Nguyen, Bradford D. Gessner, Tuya Mungun, E. Kim Mulholland, Claire von Mollendorf

**Affiliations:** a University of Melbourne, Melbourne, Australia; b Murdoch Children's Research Institute, Melbourne, Australia; c National Center for Communicable Diseases, Ministry of Health, Ulaanbaatar, Mongolia; d Ministry of Health, Ulaanbaatar, Mongolia; e Mongolian National University of Medical Sciences, Ulaanbaatar, Mongolia; f Pfizer Vaccines, Collegeville, USA; g London School of Hygiene and Tropical Medicine, London, UK

**Keywords:** PCV, Adults, Streptococcus pneumoniae, Community-acquired pneumonia, Indirect effects, Mongolia

## Abstract

**Background:**

Few studies have assessed the potential indirect effects of childhood pneumococcal conjugate vaccine (PCV) programs on the adult pneumonia burden in resource-limited settings. We evaluated the impact of childhood PCV13 immunisation on adult all-cause pneumonia following a phased program introduction from 2016.

**Methods:**

We conducted a time-series analysis to assess changes in pneumonia hospitalisation incidence at four district hospitals in Mongolia. Adults (≥18 years) that met the clinical case definition for all-cause pneumonia were enrolled. A negative binomial mixed-effects model was used to assess the impact of PCV13 introduction on monthly counts of pneumonia admissions from January 2015–February 2022. We also performed a restricted analysis excluding the COVID-19 pandemic period. All models were stratified by age and assessed separately. Additional analyses assessed the robustness of our findings.

**Findings:**

The average annual incidence of all-cause pneumonia hospitalisation was highest in adults 65+ years (62.81 per 10,000 population) and declined with decreasing age. After adjusting for the COVID-19 pandemic period, we found that rates of pneumonia hospitalisation remained largely unchanged over time. We did not observe a reduction in pneumonia hospitalisation in any age group. Results from restricted and sensitivity analyses were comparable to the primary results, finding limited evidence of a reduced pneumonia burden.

**Interpretation:**

We did not find evidence of indirect protection against all-cause pneumonia in adults following childhood PCV13 introduction. Direct pneumococcal vaccination and other interventions should be considered to reduce burden of pneumonia among older adults.

**Funding:**

Pfizer clinical research collaboration agreement (contract number: WI236621).

**Research in context**

**Evidence before this study**

We conducted a PubMed search for evidence of the paediatric 13-valent pneumococcal conjugate vaccine (PCV13) program on the burden of all-cause pneumonia in adults using the following search terms “pneumococcal”, “conjugate vaccine”, “PCV”, “adult/s” or “elderly”, “effect”, “impact”, and “all-cause pneumonia”, between 1 January 2010 and 7 March 2023. We identified publications about observational studies that evaluated the indirect effect of childhood PCV immunisation on all-cause pneumonia hospitalisation in adults (≥18 years). Two manuscripts were from the same study in Australia and analysed different follow up time points (30 months and 6.5 years), and therefore excluded the study with the shorter follow-up period. All studies used hospital admission data to assess the impact of routine PCV immunisation, with follow up periods ranging between two and 6.5 years post-introduction of routine PCV immunisation. We did not identify any studies in which PCV13 had been the first PCV option available for paediatric use. Four studies reported use of PCV7 or PCV10 prior to PCV13 introduction, four studies evaluated impact of childhood PCV10 introduction on pneumonia hospitalisations, and one study reported the effects from PCV7 introduction. Studies from Australia, Fiji, Scotland, Spain, and Ecuador did not find evidence of a reduced all-cause pneumonia burden in any adult age group, with studies from Fiji, Scotland, Spain, and Ecuador reporting increased pneumonia hospitalisation rates in older adults during their respective study periods. Two studies from the United States and Brazil found declines in all-cause pneumonia admissions in adults 18–39 years following PCV introduction (12% and ∼20%, respectively), but not in older age groups, while one study from Finland found an 15% reduction among adults ≥18 years. Additionally, one study in Hong Kong reported marginal declining trends of all-cause pneumonia in adults 65+ years; however, findings were confounded by an influenza pandemic and sensitivity analyses yielded non-significant results. There were no studies that specifically documented the effect of PCV on severe pneumonia; however, studies from Scotland and Ecuador reported changes in adult all-cause pneumonia mortality in pre- and post-PCV periods. While the study from Ecuador did not observe any reductions in mortality, the study from Scotland found >10% reduction in adult pneumonia-related deaths following PCV.

**Added value of this study**

We evaluated the impact of childhood PCV13 immunisation on adult all-cause pneumonia hospitalisation in Mongolia following a phased program introduction from 2016. PCV13 was the first infant pneumococcal vaccine used in Mongolia. This study addresses a knowledge gap for low- and middle-income countries (LMICs) and the Asia–Pacific on the impact of childhood PCV immunisation on the burden of all-cause pneumonia in adults. It is also one of the first observational studies to account for disruptions due to the COVID-19 pandemic and resulting potential biases. We did not find evidence of a protective effect on all-cause pneumonia hospitalisation rates in any adult age group following childhood PCV13 introduction. These data contribute to filling gaps in the literature on the indirect effects of PCVs in unvaccinated adults in resource-limited settings and highlight some of the challenges associated with assessing vaccine impact using surveillance data.

**Implications of all the available evidence**

Findings from this study may be relevant to other countries in the Asia–Pacific with a similar climate, socioeconomic setting, and high levels of ambient air pollution. We did not find evidence of indirect protection against all-cause pneumonia in adults in Mongolia following the introduction on PCV13. However, because effects of PCV immunisation are cumulative, it is possible that an impact could be observed with additional time. Based on the findings from this study, direct pneumococcal vaccination and other interventions aimed at reducing the pneumonia burden in older adults in Mongolia should be considered. Identification of key factors that contribute to the high rate of pneumonia in older adults could also help develop meaningful health policy strategies.

## Introduction

Community-acquired pneumonia is a major cause of morbidity in both young children and the elderly. While the widespread use of pneumococcal conjugate vaccines (PCVs) in children has considerably reduced the pneumococcal disease burden globally, *Streptococcus pneumoniae* remains a leading cause of pneumonia in older adults and those with underlying medical conditions.^1^^,^^2^ In addition to providing direct protection for vaccinated children, PCVs provide indirect protection to unvaccinated individuals by decreasing vaccine-type (VT) pneumococcal carriage rates in vaccinated children, thus reducing *S. pneumoniae* transmission in the overall population.^3^ The indirect effects from PCV accumulate over time, providing substantial protection (up to 90% reduction) from VT invasive pneumococcal disease (IPD) in the broader population within a decade.^2^ However, studies have also observed persistence of VT disease (including non-bacteraemia pneumonia and IPD) in adults, and the degree of indirect protection prior to reaching the protective threshold has varied.^3^^,^^4^

Serotype prevalence, disease burden, vaccine program factors (e.g., vaccine type, schedule, coverage rate), and other epidemiological factors vary by country and can impact the degree to which childhood PCV programs provide indirect protection for adults.^3^^,^^5^^,^^6^ Moreover, country-specific population characteristics, resource constraints, and environmental factors (e.g., air pollution) can affect the population-level impacts of PCVs.^5^^,^^6^ Despite pneumonia accounting for the highest number of deaths due to pneumococcal disease, the majority of PCV impact studies have concentrated on the IPD burden in high-income countries.^3^^,^^6^ A gap in the literature remains concerning the effects of childhood PCV immunisation on rates of adult all-cause pneumonia, particularly in low- and middle-income settings.

In 2016, the Government of Mongolia (GoM) introduced PCV13 into the national paediatric immunisation program to reduce the childhood burden of pneumonia.^7^ Carriage studies conducted in young children after PCV13 introduction found a reduction in VT carriage for both vaccinated and unvaccinated children, supporting evidence of both direct and indirect effects in this age group.^8^^,^^9^ This study aimed to evaluate the indirect impact of childhood PCV13 program introduction on the incidence of all-cause pneumonia hospitalisation in adults ≥18 years in Mongolia.

## Methods

### Study setting

In June 2016, the GoM commenced a phased introduction of routine childhood PCV13 immunisation using a 2 + 1 vaccination schedule. The program was introduced over a three-year period, beginning with two districts (Songinokhairkhan, Sukhbaatar) in the capital city of Ulaanbaatar from June 2016, an additional district in July 2017 (Bayanzurkh), and the remaining six districts (including Chingeltei) in March 2018. Catch-up campaigns were conducted for children 12–23 months in 2016 and 2017, but not in 2018. Currently, there are no pneumococcal vaccines licenced for use in adults in Mongolia.

Active, hospital-based surveillance for adult all-cause pneumonia was established in four districts (Bayanzurkh, Songinokhairkhan, Sukhbaatar, Chingeltei) of Ulaanbaatar in March 2019. In March 2020, the GoM introduced restrictions in response to the COVID-19 pandemic, with the first documented case of locally transmitted COVID-19 infection detected in November 2020.^10^ Additional details on the COVID-19 pandemic response in Mongolia can be found in Appendix p 1.

### Study design

The adult pneumonia surveillance program covered a seven-year period, and included patients enrolled retrospectively (January 2015–February 2019) and prospectively (March 2019–February 2022). Details on the study rationale, design, and eligibility criteria have been previously described.^7^

In brief, patients that met the study case definition for all-cause pneumonia were included retrospectively by conducting medical chart reviews. From March 2019, patients were enrolled prospectively if they met the study eligibility criteria and data were collected through reviewing medical charts and patient interviews. Patients were ineligible for inclusion if they had not resided in one of the participating districts for three or more months or had been readmitted to the hospital with pneumonia within 14 days of their previous admission. Additionally, during the COVID-19 pandemic period, all patients with respiratory symptoms were tested for SARS-CoV-2 at admission using rapid antigen testing and results were confirmed by quantitative real-time polymerase chain reaction. Patients with a positive SARS-CoV-2 result were excluded from this study and were transferred to a COVID-19 treatment facility.^7^

We defined all-cause pneumonia as a patient admission with clinician-diagnosed pneumonia and with ≥2 clinical symptoms associated with pneumonia. Severe pneumonia was defined as a pneumonia admission with 1) an intensive care unit stay, 2) death during hospitalisation, or 3) with ≥2 of the following symptoms indicative of severity: impaired conscious state, ≥30 breaths per minute, hypotension, or hypoxemia.^7^

Chest radiographs (CXRs) were collected from patients enrolled prospectively and results were reported by two independent radiologists. We defined primary-endpoint pneumonia as the presence of focal endpoint consolidation or pleural effusion in the lateral pleural space spatially associated with a pulmonary parenchymal infiltrate, or if the effusion obliterated enough of the hemithorax to obscure an opacity.^7^ We were unable to collect CXRs from one district hospital from July 2021–January 2022 due to a non-functioning X-ray machine. More details on the radiological pneumonia case definition and data collection methodology can be found in the study protocol.^7^

### Oxford COVID-19 government response tracker (OxCGRT) stringency index

To account for changes in the stringency of nonpharmaceutical intervention (NPI) policies implemented during the COVID-19 pandemic, we included the OxCGRT stringency index in our analysis. The OxCGRT collected data on national COVID-19 pandemic policy response indicators for most countries and aggregated the indicators into standardised indices.^11^ The stringency index provides a composite measure for nine of the indicators, including school or workplace closures and restrictions on population movement. The index is scaled from 0 to 100 (100 = strictest) and has been used to estimate the impacts of government COVID-19 policies on a number of health outcomes. Several countries have reported strong associations between an increased stringency of NPIs and declines in respiratory infections.^12^, ^13^, ^14^ For our analysis, daily stringency index values were averaged by calendar month from March 2020. All months prior to March 2020 were assigned a stringency index of 0.

### Statistical analysis

Case characteristics were described, and annual crude incidence rates with 95% Poisson confidence intervals were calculated for all pneumonia admissions. Annual, district-specific population denominators were provided by the National Statistics Office of Mongolia. We calculated the average annual incidence rates of pneumonia hospitalisation, overall and by age group, for the seven-year study period (2015–2021).

A negative binomial mixed-effects model was used to assess the impact of PCV13 introduction on monthly counts of all-cause pneumonia and severe pneumonia from January 2015–February 2022. A negative binomial distribution was chosen due to overdispersion in the data. District-specific vaccine introduction months were used to indicate pre- and post-PCV13 periods, where the pre-PCV13 period was defined as January 2015 until the month prior to PCV13 introduction, and the post-PCV13 period was defined as the month of PCV13 introduction through February 2022. We included a linear term for time (in months) to adjust for underlying secular trends and calendar month to adjust for seasonality.^15^ Calendar month was selected for this model instead of Fourier terms because calendar month resulted in improved model fit as measured by the Akaike's Information Criterion (AIC). Indicator variables were used to adjust for the impacts of the COVID-19 pandemic, including the average monthly stringency index value as a time-varying covariate and a binary variable to capture the pandemic period (‘0’ pre-pandemic period and ‘1’ pandemic period).^11^^,^^16^ Log-transformed population denominators were included as an offset. District of hospitalisation was included as a random-effect. Models were stratified into age groups (18–25, 26–45, 46–64, and 65+ years). Because PCV13 coverage was high (>95%) among targeted age groups in the four districts, we assumed a uniform vaccine impact.^17^ Model fit was evaluated using the AIC.

We conducted a subgroup analysis that restricted the surveillance data to patients hospitalised prior to the pandemic period (January 2015–February 2020) to investigate whether our assumptions about pandemic-related confounders were appropriate and to test model sensitivity. All pre-pandemic covariates and assumptions from the primary analysis remained the same.

As a robustness check, we conducted a two-stage interrupted time series analysis to evaluate changes in pneumonia hospitalisation rates following PCV13 introduction compared with the pre-PCV13 period.^18^ Monthly counts of pneumonia hospitalisation were used to evaluate step (immediate) changes and slope (gradual) changes over time following PCV13 introduction for each district. In the first stage, models were stratified by age group and district of admission using a negative binomial distribution to allow for overdispersion. Because the stringency index may not fully capture the impacts of the pandemic and NPIs in our study population, we also adjusted for the change in trend (slope) during the pandemic period. Accordingly, we included district-specific interruption timepoints based on the month and year of PCV13 introduction in the model to account for changes in pneumonia rates over time, while also including a binary variable to account for the onset of the COVID-19 pandemic period (‘0’ pre-pandemic period, ‘1’ pandemic period).^11^^,^^16^ We also used a linear term for time (in months) to capture long-term trends, calendar month to adjust for seasonality, and log-transformed population denominators were included as an offset.^15^ In the second stage, district-specific estimates were pooled by age group for pneumonia incidence using a random-effect meta-analysis with restricted maximum likelihood methodology.^18^ Cases of severe pneumonia were not assessed separately due to low case numbers.

Two additional sensitivity analyses were performed to assess the robustness of our findings. First, we added a lag period at three months, six months, and 12 months post-PCV13 introduction to investigate if there was a time lag prior to observing an impact. Second, to validate the appropriateness of our assumptions for when we would expect to see the impacts of the COVID-19 pandemic in our data, we employed a breakpoint analysis to identify structural changes in the time series for monthly pneumonia hospitalisations.^16^^,^^19^ Additional details on the analyses used for sensitivity analyses are found in Appendix p 1.

Data were analysed using Stata version 16.1 and R version 4.1.1.

### Ethical review

The study was approved by the Ethics Committee for Health Research of Ministry of Health, Mongolia and the Royal Children's Hospital, Melbourne (HREC 38045). Written informed consent was obtained from all study participants included in prospective data collection.

### Role of funding source

This study was conducted as a collaboration between Murdoch Children's Research Institute (MCRI) and Pfizer (contract number: WI236621). MCRI is the study sponsor.

## Results

**Fig. 1:**
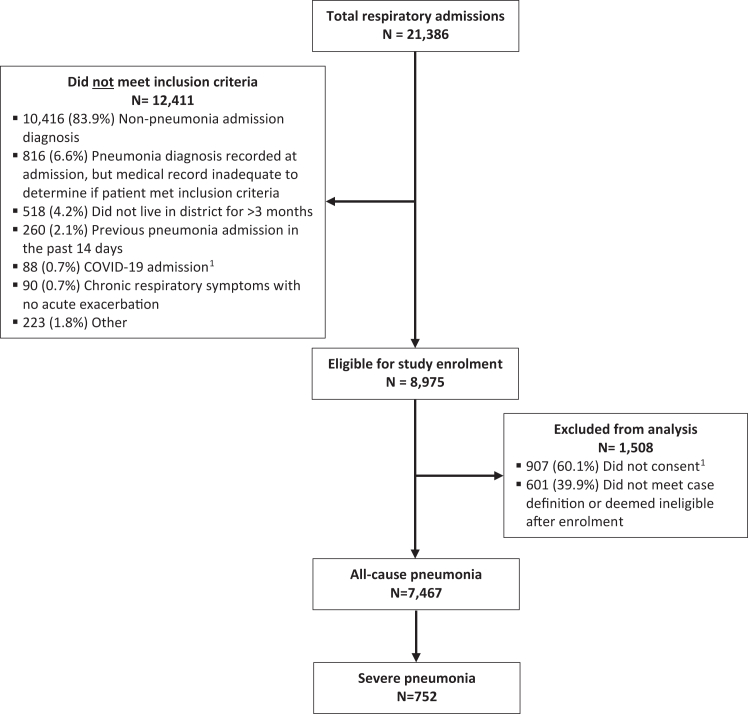
**Inclusion of study participants with respiratory-related admissions at four sentinel hospitals in Mongolia, January 2015–February 2022**. ^1^Indicates that the exclusionary reason was only relevant during the prospective data collection period (March 2019–February 2022).

**Fig. 2:**
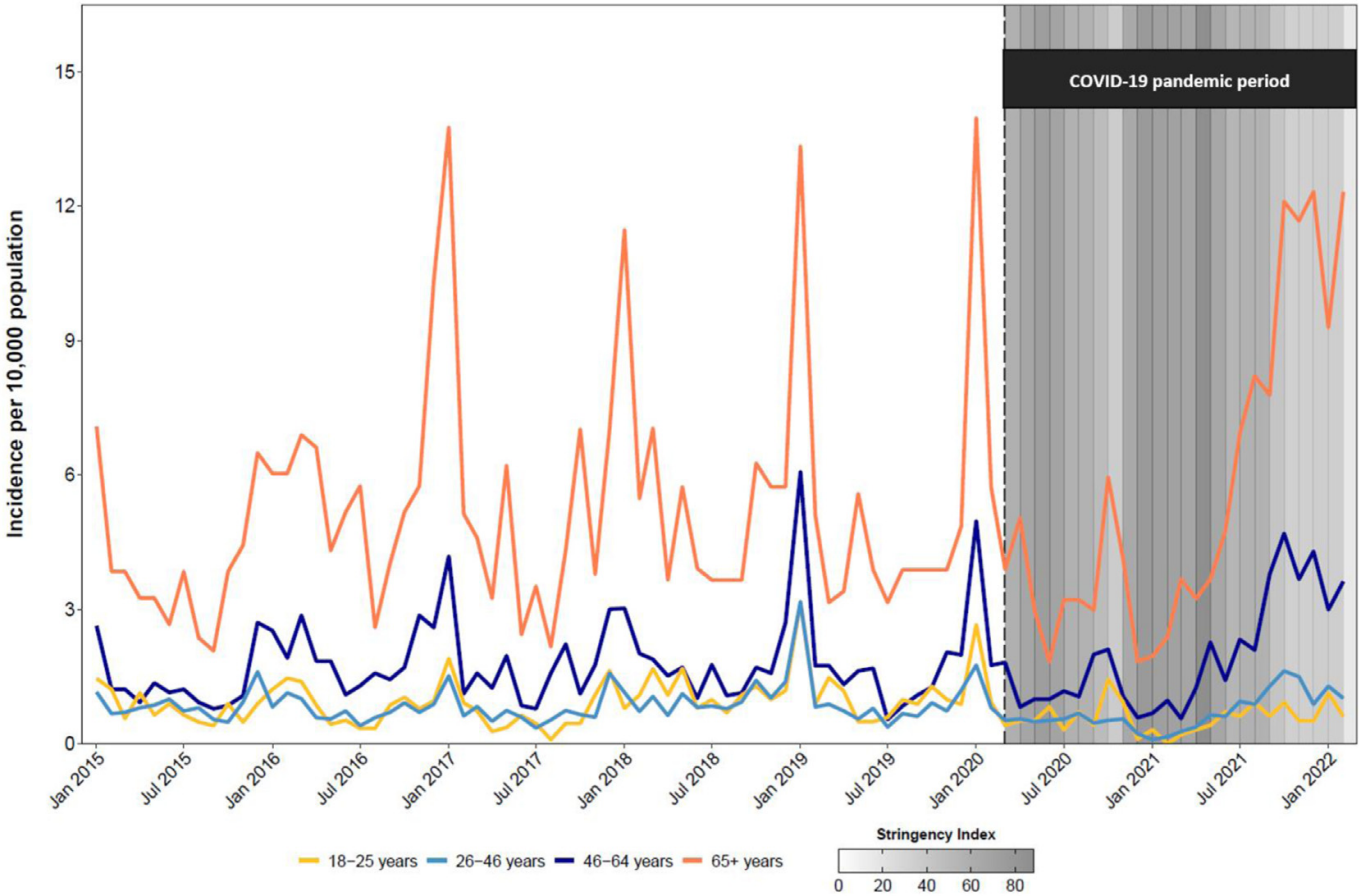
I**ncidence of all-cause pneumonia hospitalisation, by age group**. Dashed line indicates beginning of pandemic period (March 2020). Average monthly OxCGRT stringency index values indicate stringency of government policies implemented during pandemic period, scaled from 0 to 100 (strictest).

In total, 21,386 respiratory admissions were screened at the four district hospitals between January 2015 and February 2022 (Fig. 1). Of 7,467 admissions that met the case definition for all-cause pneumonia, 752 (10.1%) were classified as severe pneumonia. Almost half (49.2%) of the patients with pneumonia had at least one underlying medical condition, and 89 (1.2%) patients died during hospitalisation (Appendix p 2). Pneumonia hospitalisation rates followed distinct seasonal patterns and were highest in Mongolia's winter months (November–March) except for the 2020–2021 winter, which occurred during the COVID-19 pandemic period (Fig. 2).

### Burden of all-cause pneumonia

From 2015 to 2021, the average annual incidence of all-cause pneumonia hospitalisation for all adults ≥18 years was 16.14 admissions per 10,000 population (Table 1). Average pneumonia hospitalisation rates were lowest in the 18–25-year-old and 26–45-year-old age groups (10.17 and 9.79 admissions per 10,000 population, respectively). In adults 65+ years, the average incidence was 62.81 admissions per 10,000 population—a six-fold increase from the two youngest age groups. While annual incidence rates varied slightly over time, in general, the incidence was lowest in 2015 and increased annually until 2020, where rates declined in all age groups. Compared to the pre-pandemic period (2015–2019), hospitalisation rates for all-cause pneumonia decreased in age groups 45 years and younger and increased in age groups >45 years during the COVID-19 pandemic period (2020–2021) (Appendix p 3–4).

**Table 1:** Population-based incidence of adults hospitalised with all-cause pneumonia and severe pneumonia, per 10,000 population, Mongolia, 2015–2021.

	2015		2016		2017		2018		2019		2020		2021		7-year average (2015–2021)	
	n	IR (95% CI)	n	IR (95% CI)	n	IR (95% CI)	n	IR (95% CI)	n	IR (95% CI)	n	IR (95% CI)	n	IR (95% CI)	n	IR (95% CI)
**All-cause pneumonia**																
All 18+ years	825	13.49 (12.58–14.44)	997	15.91 (14.94–16.93)	972	15.22 (14.28–16.21)	1,117	17.65 (16.63–18.71)	1,114	17.47 (16.46–18.52)	912	14.29 (13.37–15.24)	1,221	18.80 (17.76–19.89)	**1,023**	**16.14 (15.17–17.16)**
18–25 years	120	9.67 (8.01–11.56)	118	10.16 (8.41–12.17)	99	8.98 (7.30–10.93)	135	13.27 (11.13–15.71)	135	13.22 (11.08–15.64)	95	9.69 (7.84–11.84)	59	6.02 (4.59–7.77)	**109**	**10.17 (8.35–12.27)**
26–45 years	321	10.25 (9.16–11.44)	295	8.97 (7.98–10.06)	312	9.23 (8.23–10.31)	394	11.80 (10.67–13.03)	375	11.43 (10.30–12.65)	248	7.62 (6.70–8.63)	303	9.23 (8.22–10.33)	**321**	**9.79 (8.75–10.92)**
46–64 years	225	15.99 (13.97–18.22)	345	23.48 (21.07–26.10)	327	21.36 (19.11–23.80)	335	21.05 (18.85–23.42)	365	21.95 (19.76–24.32)	330	19.29 (17.26–21.49)	495	27.96 (25.55–30.54)	**346**	**21.74 (19.51–24.15)**
65+ years	159	46.94 (39.92–54.82)	239	68.66 (60.23–77.94)	234	63.15 (55.32–71.78)	253	65.95 (58.07–74.59)	239	57.96 (50.85–65.80)	239	54.73 (48.01–62.13)	364	78.67 (70.79–87.18)	**247**	**62.81 (55.22–71.15)**
**Severe pneumonia**																
All 18+ years	86	1.41 (1.12–1.74)	105	1.68 (1.37–2.03)	106	1.66 (1.36–2.01)	97	1.53 (1.24–1.87)	110	1.72 (1.42–2.08)	142	2.22 (1.87–2.62)	93	1.43 (1.16–1.75)	**106**	**1.67 (1.37–2.02)**
18–25 years	4	0.32 (0.09–0.82)	4	0.34 (0.09–0.88)	3	0.27 (0.06–0.80)	1	0.10 (0–0.55)	7	0.69 (0.28–1.41)	9	0.92 (0.42–1.74)	2	0.20 (0.02–0.74)	**4**	**0.37 (0.10–0.96)**
26–45 years	26	0.83 (0.54–1.22)	23	0.70 (0.44–1.05)	21	0.62 (0.38–0.95)	18	0.54 (0.32–0.85)	28	0.85 (0.57–1.23)	33	1.01 (0.70–1.42)	12	0.37 (0.19–0.64)	**23**	**0.70 (0.44–1.05)**
46–64 years	28	1.99 (1.32–2.88)	36	2.45 (1.72–3.39)	45	2.94 (2.14–3.93)	34	2.14 (1.48–2.98)	37	2.23 (1.57–3.07)	56	3.27 (2.47–4.25)	37	2.09 (1.47–2.88)	**39**	**2.45 (1.74–3.35)**
65+ years	28	8.27 (5.49–11.95)	42	12.07 (8.70–16.31)	37	9.99 (7.03–13.76)	44	11.47 (8.33–15.40)	38	9.22 (6.52–12.65)	44	10.08 (7.32–13.53)	42	9.08 (6.54–12.27)	**39**	**9.92 (7.05–13.56)**

Bold text refers to the averaged annual incidence from 2015 to 2021, per 10,000 population. IR, Incidence rate; CI, Confidence Interval.

The average annual incidence per 10,000 population of severe pneumonia was 1.67 for all adults ≥18 years for the seven year period (Table 1). Average severe pneumonia rates were lowest in the younger age groups and increased with age, reaching 9.92 admissions per 10,000 population in adults 65+ years. For all age groups, we observed similar rates of severe pneumonia hospitalisations annually, although rates marginally increased in 2020 for age groups <65 years. Compared to the pre-pandemic period, the incidence of severe pneumonia during the pandemic period increased for adults 18–25 years and 46–64 years, slightly decreased for adults 65+ years, and remained the same for those aged 26–46 years (Appendix p 3–4).

### Radiological pneumonia

We obtained CXRs for 2,543 of 3,178 (80.0%) patients enrolled with all-cause pneumonia from March 2019–February 2022. Of these, 211 (8.3%) were uninterpretable and excluded from analysis. In total, 722 (31.0%) of 2,332 patients were diagnosed with primary-endpoint pneumonia and 461 (19.8%) were diagnosed with other infiltrates (Appendix p 2). Of 322 enrolled patients with severe pneumonia, 253 (78.6%) had a CXR performed; 30 (11.9%) CXRs were excluded because they were uninterpretable. In total, 127 (57.0%) patients with severe pneumonia were diagnosed with primary-endpoint pneumonia and 35 (15.7%) were diagnosed with other infiltrates (Appendix p 2). Of 32 patients that died during hospitalisation and had a CXR result, 20 (62.5%) were diagnosed with primary-endpoint pneumonia.

### Indirect PCV13 impact

**Fig. 3:**
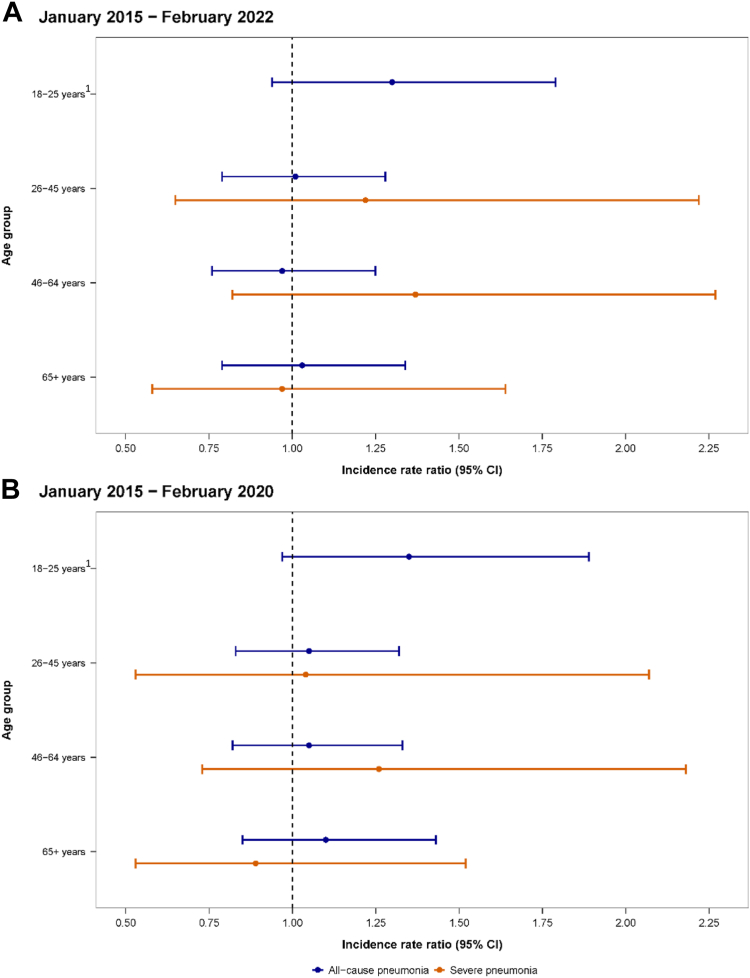
**Incidence rate ratios (IRRs) of pneumonia hospitalisation incidence in adults comparing pre- and post-PCV13 introduction, by age and disease severity, by (A) full surveillance period (January 2015–February 2022) and (B) excluding COVID-19 pandemic period (January 2015–February 2020)**. IRRs calculated using negative binomial mixed-effects model to assess the indirect impact of PCV13 introduction on monthly counts of all-cause pneumonia and severe pneumonia, adjusting for seasonality, secular trends, and log-transformed population denominators as an offset. District of hospitalisation included as a random-effect. (A) additionally adjusted for the COVID-19 pandemic period. 1 Severe pneumonia not calculated due to small sample size.

Results from our analyses on the impact of PCV13 introduction on all-cause pneumonia hospitalisation are shown in Fig. 3. From pre-PCV13 to post-PCV13 periods, we did not observe any statistically significant reductions in all-cause pneumonia or severe pneumonia hospitalisations in any age group over the seven-year period (Fig. 3a). Analyses restricted to the pre-pandemic period also found no evidence of a change in all-cause pneumonia or severe pneumonia hospitalisations in any age group (Fig. 3b).

Data from the complete study period and restricted period revealed similar incidence rate ratios (IRRs) for all-cause pneumonia hospitalisation across stratified age groups. While not statistically significant, we observed a trend towards an increased burden in all-cause pneumonia hospitalisations following PCV13 introduction in those aged 18–25 years (IRR: 1.30; 95% CI: 0.94–1.79) from 2015 to 2022. A similar trend was identified when analyses were restricted to the pre-pandemic period (IRR: 1.35; 95% CI: 0.97–1.89). For age groups >25 years, all-cause pneumonia hospitalisation rates remained largely unchanged following the introduction of childhood PCV13 immunisation.

### Two-stage interrupted time series

**Fig. 4:**
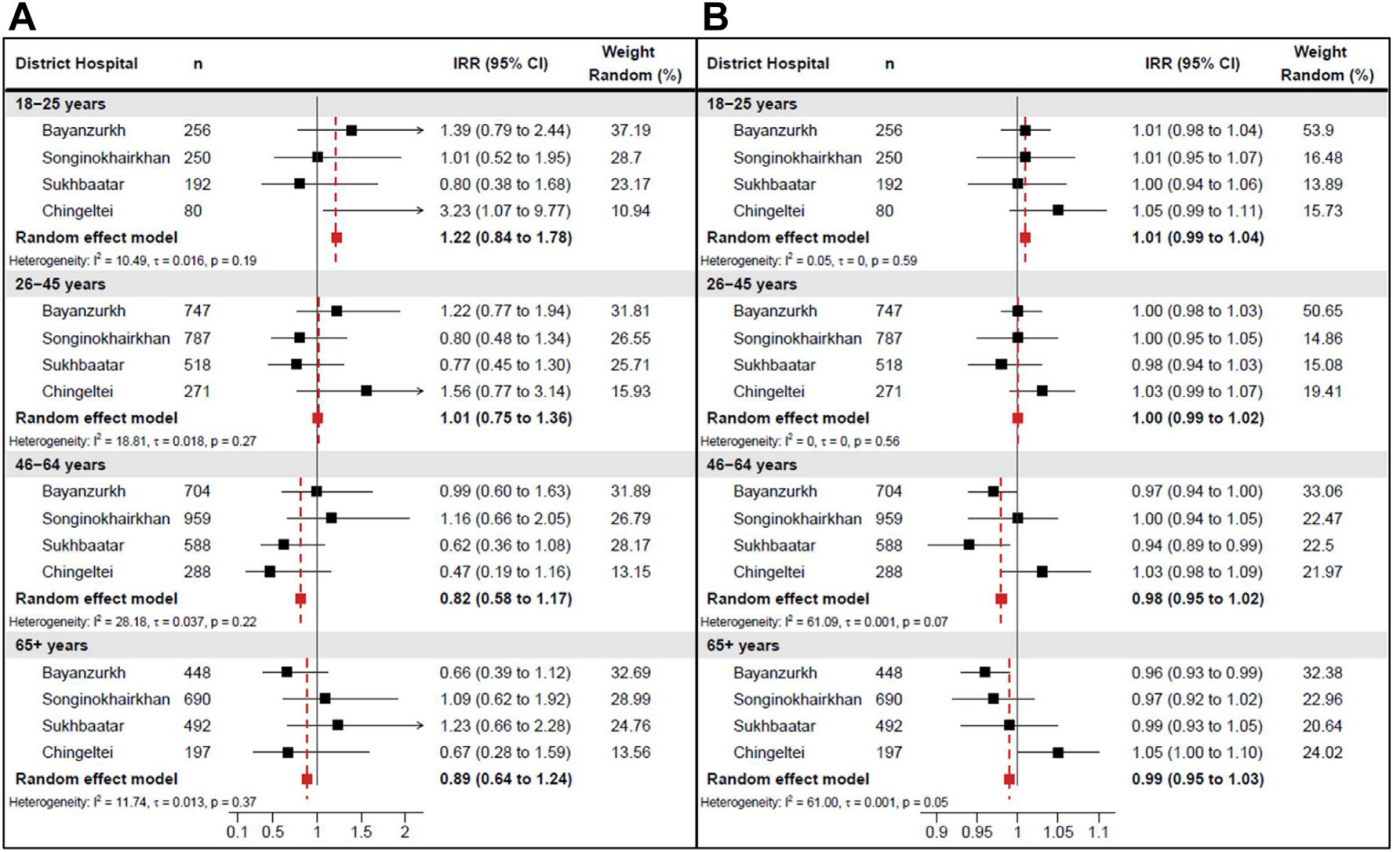
**District-specific incidence rate ratios (IRRs) for adult all-cause pneumonia admissions measuring (A) step and (B) slope PCV13 impact on adult pneumonia following introduction of Mongolia's childhood PCV13 program, by the age group, January 2015–February 2022**. Estimated district-specific IRRs were calculated using an interrupted time series model that allows for (A) step and (B) slope change in monthly pneumonia incidence from the point of childhood PCV13 introduction. Model adjusted for secular time, seasonality, the COVID-19 pandemic period, and log-transformed population denominators were included as an offset. To adjust for the COVID-19 pandemic period, a binary pre-/post-pandemic variable and a variable to account for gradual pandemic-related changes in trend were included.

District-specific and pooled estimates are presented in Fig. 4. Results from the pooled analyses showed no significant changes in all-cause pneumonia hospitalisation rates immediately following PCV13 introduction in any age group, though some (non-significant) reductions were detected in the 46–64 year- and 65+ year-old age groups (Fig. 4a). Additionally, pneumonia hospitalisation trends did not change over the study period for any age group (Fig. 4b). While there were some variations between districts in each age group, changes in trend following PCV13 introduction were largely similar over time. Results from pooled analyses found minimal changes in slope for all age groups, indicating that pneumonia hospitalisation trends remained unchanged over time.

### Sensitivity analysis

For all age groups, results from the sensitivity analyses using time lags did not materially change our estimates from the primary analysis (Appendix p 3). Results from the breakpoint analysis identified three breakpoints in November 2017, February 2020, and February 2021, aligning with a period of high influenza burden, followed by changes during the pandemic period, supporting our assumption that pandemic-related disruptions likely biased our results (Appendix p 5).

## Discussion

In this study, we investigated the burden of hospitalised all-cause pneumonia in adults before and after PCV13 introduction into the paediatric immunisation program. Over the seven-year study period, we did not observe a clear impact of PCV13 on the all-cause pneumonia burden in any adult age group, after adjusting for the impacts of COVID-19 pandemic and secular trends. Our study highlights some of the challenges associated with assessing vaccine impact using surveillance data.

The magnitude of protection against pneumonia attributed to childhood PCV immunisation varies. The United States and Finland have consistently reported a reduced incidence of adult all-cause pneumonia following the introduction of childhood PCV programs.^20^^,^^21^ Conversely, studies from Fiji, Canada, and Australia have found limited evidence of a reduced all-cause pneumonia burden in adults after childhood PCV introduction.^22^, ^23^, ^24^ Several factors could contribute to heterogeneity in the observed PCV impact on all-cause pneumonia across settings and explain our findings. First, despite high PCV13 coverage in the study districts, a longer follow-up period might be required to see an effect. In the US and Finland, decreasing trends in pneumonia hospitalisations were seen within 5–6 years after PCV introduction, but it can take almost a decade for the protective effects to spread to a whole population.^2^^,^^20^^,^^21^ This hypothesis is undermined by a Canadian study that found no reductions in pneumonia rates in older adults 15 years after sequential PCV7 and PCV13 introduction.^24^ Second, pneumococcal serotype distribution and antibiotic susceptibility can vary widely across settings and likely contribute to the heterogeneity of our results. While rates of pneumococcal disease have declined, globally, with the introduction of PCVs, many countries have observed persistence of serotypes 19F, 19A, and 3 and increased carriage of non-VT serotypes—though the degree of PCV impact on serotype distribution varies across settings.^6^^,^^25^^,^^26^ Moreover, data from six countries in Asia found high prevalence of antimicrobial resistance in adults 50+ years, with multidrug resistant carriage rates ranging from 3.1% (Philippines) and 76.0% (China).^25^ In Mongolia, following PCV13 introduction, carriage of VT serotypes significantly declined in vaccine-eligible toddlers as well as infants too young to be vaccinated, providing some evidence of indirect effects.^8^ However, carriage rates of non-VT serotypes in young children have increased after PCV13 introduction, and studies have reported high prevalence (>80%) of antimicrobial resistance.^8^^,^^9^ It is possible that rates of persistent VT serotypes or antimicrobial-resistant non-VT serotypes attenuated the indirect effects of PCV13 in Mongolia.^6^^,^^27^ Alternatively, the proportion of pneumonia cases caused by VT pneumococci may not have been large enough to find a statistically significant impact. Results from nasopharyngeal and urine samples collected during the prospective study period will provide some insight into the pneumococcal pneumonia burden and serotype distribution in Mongolia, once the results become available. Other factors, such as differences in vaccine programs, healthcare utilisation, admission practices, population risk factors, and surveillance methodology, further contribute to the variance in indirect PCV effectiveness on adult pneumonia. The relatively low mortality in our study might also be reflective of these differences.

Our analyses were subject to two major confounders that could have influenced our findings: a national influenza epidemic in Mongolia during the 2018–2019 winter and the COVID-19 pandemic.^28^ Annual rates for influenza-associated severe acute respiratory infection increased steadily between 2013 and 2018 in Mongolia, and a nationwide epidemic was declared in the winter of 2018–2019.^28^^,^^29^ Because influenza is a major cause of pneumonia and is also a risk factor for *S. pneumoniae* infection, it is possible that the high rates of influenza in Mongolia could have reduced the indirect effect of PCV13 on pneumonia hospitalisation rates in adults.^30^ Additionally, the onset of the COVID-19 pandemic had profound impacts on healthcare systems, population mobility behaviours, and communicable disease transmission patterns.^13^^,^^31^ We aimed to minimise pandemic-related bias by excluding patients with a positive SARS-CoV-2 result and by conducting additional analyses to ensure our results were robust, though it is possible that some residual confounding remained.^16^ While the stringency index accounted for time-varying changes in NPI stringency, values were aggregated at the country-level and did not account for changes in healthcare seeking behaviours, local containment measure policies, or socio-demographic variations in mobility patterns.^11^ However, because results from the interrupted times series and restricted analyses were comparable to our primary analysis, it is unlikely that the inclusion of other unmeasured factors would alter our findings. Other potential confounders, such as air pollution levels, were not included in this analysis and may have influenced pneumonia hospitalisation rates.^32^ Consequently, the findings of this analysis should be carefully interpreted.

### Limitations

This study had several important limitations in addition to the ones previously mentioned. First, it is possible that some pneumonia cases were not detected from hospital admissions logs for patients enrolled retrospectively due to missing information. However, proportions of severe pneumonia admissions and deaths were similar between patients enrolled retrospectively and prospectively, making it less likely that a large number of cases were missed. Second, the observed incidence rates from this evaluation likely underestimated the total burden of pneumonia. Self-treatment and financial barriers are important factors that impede patients from seeking inpatient care and could have motivated some patients to seek care from primary or secondary outpatient facilities instead.^33^ Conversely, use of privatised hospitals is increasing in Mongolia, particularly for high-income households, despite greater out-of-pocket costs.^33^ However, we were unable to obtain data from outpatient clinic visits or inpatient admissions at private hospitals and do not include cases from these settings in our estimates. Third, we did not estimate the incidence of pneumococcal pneumonia or changes in serotype prevalence over time because samples were only collected between March 2019 and February 2022 and serotyping results are not yet available. Pneumococcal serotyping results will provide additional insights into the serotype prevalence and distribution of pneumococcal disease in this population and could inform health policy strategies aimed at reducing the adult pneumonia burden. Finally, findings from this study may not be translatable to other provinces (aimags) in Mongolia during the study period. Ulaanbaatar is more densely populated and was more heavily impacted by the COVID-19 pandemic than other provinces, which could have resulted in different patterns of disease transmission.^10^^,^^31^ However, findings from this analysis may be relevant to other countries in the region with a similar climate, socioeconomic setting, or high levels of ambient air pollution.

### Conclusions

Following the introduction of the childhood PCV13 program in Mongolia, we did not find evidence of an indirect, protective effect against adult all-cause pneumonia. Future studies should consider assessing the impact of serotype replacement and defining the impact of childhood PCV programs on pneumococcal pneumonia and radiological pneumonia in adults. Further, identifying key factors that contribute to the pneumonia incidence in older adults could help develop meaningful health policy strategies to reduce the rate of hospitalised pneumonia. Given the high burden of all-cause pneumonia in older adults and limited indirect impact from paediatric immunisation, direct pneumococcal vaccination and other targeted interventions should be considered.

## Contributors

CvM and EKM conceived and designed the original study. CvM, EKM, TM, BT, DN, EMD, BDG, and KEA were responsible for study oversight. MU, BS, DL, PB, and TM collected and managed the data. KF, CvM, and CDN contributed to the analysis plan. KF conducted the data cleaning and formal analysis, created the tables and figures, and drafted the manuscript. CvM, TM, EMD, BDG, KEA, ACG, JdC, and MdC supported interpretation of the data. CvM verified the data and analysis. All authors were involved in review, editing, and revision of the manuscript. All authors approved the final version of the manuscript.

## Data sharing statement

All relevant data are within the paper and the Supplementary information files.

## Declaration of interests

EKM and CVM are lead investigators of this study through Murdoch Childrens Research Institute, which was funded by Pfizer. MU, BS, DL, PB, BT, DN, CDN, and TM are investigators on this collaborative research project funded by Pfizer. EMD, KEA, and BDG are employees of Pfizer and own Pfizer stock or stock options. The other authors have no relevant conflicts of interest to declare. This study is a Murdoch Childrens Research Institute-sponsored study which is investigator-led, and funded under a collaborative agreement by Pfizer Inc.
