# Supplementary material for: Impact of childhood 13-valent pneumococcal conjugate vaccine introduction on adult pneumonia hospitalisations in Mongolia: a time series analysis
Source: Lancet Reg Health West Pac. 2023 Dec 11;44:100983. doi: 10.1016/j.lanwpc.2023.100983 (PMC10733683; doi:10.1016/j.lanwpc.2023.100983)
Supplement: Supplementary Figures and Tables [file mmc1.pdf]

SUPPLEMENTARY WEB APPENDIX

Supplement to: Impact of childhood 13-valent pneumococcal conjugate vaccine introduction on adult pneumonia hospitalisations in Mongolia: a time series analysis

Supplementary Methods

Study setting and design

Mongolia began COVID-19 pandemic preparations in late January 2020, prior to officially closing the country's borders and introducing government restrictions in March 2020[1,2]. Routine sentinel surveillance was established by the end of February 2020, and all repatriated citizens, suspected or probable cases, and close contacts were tested for SARS-CoV-2 by quantitative real-time polymerase chain reaction[1]. The first documented case of locally transmitted COVID-19 infection was detected on 10 November 2020, with active surveillance introduced into hospitals and other settings shortly thereafter[2]. Between November 2020 and February 2022, four waves of disease were reported, peaking in April 2021, June 2021, September 2021, and January 2022, respectively[3]. As a result, the Government of Mongolia (GoM) implemented four strict lockdowns in Ulaanbaatar to slow the transmission of COVID-19 (11 November—13 December 2020; 23 December 2020—10 January 2021; 11 February—22 February 2021; 10 April—7 May 2021)[4]. Additional information further describing Mongolia's experience during the COVID-19 pandemic, including the GoM pandemic response, lessons learned, as well as social, economic, and health impacts can be found elsewhere[2,5].

We planned to account for changes in secular trends between retrospective and prospective periods by including a control in our analysis[6]. Admission logs were reviewed and the total number of hospitalisations due to cardiovascular disease (CVD) and renal disease were aggregated monthly by admissions diagnosis, age group, and district hospital for the entire study period. However, both CVD and renal disease hospitalisations experienced steep, downward trends from November 2020 (appendix p 4). Because declines in CVD and renal disease hospitalisations coincided with the introduction of widespread COVID-19 transmission, changes were attributed to pandemic-related factors (e.g., changes in hospital admission practices, health behaviours, economics). Since all-cause pneumonia hospitalisations did not experience similar downward trends, including these control conditions in the analysis would likely have biased the vaccine effects estimates towards the null.

Statistical analysis

As a sensitivity analysis we used a breakpoint analysis for all adult (≥18 years) hospital admissions with all-cause pneumonia to identify population-level shifts in pneumonia hospitalisation trends within the time series[7]. Unlike interrupted time series, breakpoint detection requires fewer assumptions and does not define an intervention in its analysis. Linear regression was used to estimate breakpoints through minimizing the residual sum of squares using the *strucchange* package in R[7]. Seasonal trends were adjusted using seasonal and trend decomposition using Loess methodology and the *forecast* package[8,9]. The number of breakpoints was detected by Bayesian Information Criterion[10].

Supplementary Tables & Figures

Supplementary Table 1. Characteristics of adults hospitalised with community-acquired pneumonia, by retrospective and prospective data collection method

	All pneumonia admissions N = 7467 (%)	Retrospective period (Jan 2015—Feb 2019) N = 4289 (%)	Prospective period (Mar 2019—Feb 2022) N = 3178 (%)
**Age group**			
18-25 years	778 (10.4)	512 (12.0)	265 (8.3)
26-45 years	2323 (31.1)	1453 (33.9)	870 (27.4)
46-64 years	2539 (34.0)	1362 (31.8)	1177 (37.0)
65+ years	1827 (24.5)	961 (22.4)	866 (27.3)
**Gender**			
Female	4314 (57.8)	2506 (58.4)	1808 (56.9)
Male	3153 (42.2)	1783 (41.6)	1370 (43.1)
**Hospital district**			
Bayanzurkh	2155 (28.9)	1249 (29.1)	906 (28.5)
Songinokhairkhan	2686 (36.0)	1537 (35.8)	1149 (36.2)
Sukhbaatar	1790 (24.0)	1044 (24.3)	746 (23.5)
Chingeltei	836 (11.2)	459 (10.7)	377 (11.9)
**≥1 underlying medical condition[1]**	3673 (49.2)	1985 (46.3)	1688 (53.1)
**Severe pneumonia**	752 (10.1)	430 (10.0)	322 (10.1)
**Disease outcome**			
Death	89 (1.2)	46 (1.1)	43 (1.4)
Recovered w/o sequelae	3418 (45.8)	1846 (43.0)	1572 (49.5)
Recovered w/ sequelae[2]	3777 (50.6)	2311 (53.9)	1466 (46.1)
Transferred to another hospital[3]	138 (1.9)	75 (1.8)	63 (2.0)

[1]Includes asthma, chronic obstructive pulmonary disease (COPD)/emphysema, tuberculosis, cirrhosis/liver failure, coronary artery disease, hypertension, heart failure, chronic renal failure, and diabetes; [2]Any pneumonia-related symptoms or any subsequent complications related to their hospitalisation at the time of discharge, based on the clinical judgement of treating physicians; [3]Health outcome after transfer not available

Supplementary Table 2. Radiology results and patient characteristics for adults enrolled with pneumonia, by chest radiograph (CXR) result, March 2019— February 2022

	Total N (%)	Primary-endpoint pneumonia N (%)	Other infiltrates[1] N (%)	Negative CXR[2] N (%)
**All-cause pneumonia**	**N = 2332**	**N = 722**	**N = 461**	**N = 1149**
Age group				
18-25 years	205 (8.8)	49 (6.8)	21 (4.6)	135 (11.8)
26-45 years	634 (27.2)	174 (24.1)	93 (20.2)	367 (31.9)
46-64 years	860 (36.9)	282 (39.1)	187 (40.6)	391 (34.0)
65+ years	633 (27.1)	217 (30.1)	160 (34.7)	256 (22.3)
Gender				
Female	1327 (56.9)	307 (42.5)	242 (52.5)	778 (67.7)
Male	1005 (43.1)	415 (57.5)	219 (47.5)	371 (32.3)
≥1 underlying medical condition[3]	1233 (52.9)	396 (54.9)	266 (57.7)	571 (49.7)
**Severe pneumonia**	**N = 223**	**N = 127**	**N = 35**	**N = 61**
Age group				
18-25 years	6 (2.7)	2 (1.6)	0 (0)	4 (6.6)
26-45 years	49 (22.2)	27 (21.3)	7 (20.0)	15 (24.6)
46-64 years	88 (39.5)	61 (48.0)	11 (31.4)	16 (26.2)
65+ years	80 (35.9)	37 (29.1)	17 (48.6)	26 (42.6)
Gender				
Female	98 (44.0)	49 (38.6)	14 (40.0)	35 (57.4)
Male	125 (56.1)	78 (61.4)	21 (60.0)	26 (42.6)
≥1 underlying medical condition[3]	149 (66.8)	81 (63.8)	26 (74.3)	42 (68.9)

[1]Defined as other patchy interstitial infiltrates without a pleural effusion or focal endpoint consolidation; [2]Negative for radiological pneumonia; [3]Includes asthma, COPD/emphysema, tuberculosis, cirrhosis/liver failure, coronary artery disease, hypertension, heart failure, chronic renal failure, and diabetes

Supplementary Table 3. Crude annual population-based incidence and incidence rate ratios (IRRs) of adults hospitalised with all-cause pneumonia and severe pneumonia, by study years prior to, and during the COVID-19 pandemic per 10,000 population, 2015—2021

	**Pre-pandemic period** *(2015—2019)* **IR (95% CI)**	**Pandemic period** *(2020—2021)* **IR (95% CI)**	**IRR (95% CI)**
**All-cause pneumonia**			
All ≥18 years	*15.96 (15.53-16.41)*	*16.56 (15.87-17.28)*	*1.04 (0.99-1.09)*
18-25 years	10.95 (10.10-11.86)	7.86 (6.66-9.20)	0.72 (0.60-0.86)
26-45 years	10.34 (9.85-10.84)	8.43 (7.74-9.16)	0.82 (0.74-0.90)
46-64 years	20.84 (19.83-21.89)	23.70 (22.11-25.37)	1.14 (1.04-1.24)
65+ years	60.65 (57.15-64.30)	67.04 (61.80-72.62)	1.11 (1.00-1.22)
**Severe pneumonia**			
All ≥18 years	*1.60 (1.46-1.75)*	*1.83 (1.60-2.07)*	*1.14 (0.97-1.33)*
18-25 years	0.34 (0.21-0.54)	0.56 (0.28-1.00)	1.64 (0.70-3.62)
26-45 years	0.71 (0.58-0.85)	0.69 (0.50-0.92)	0.97 (0.67-1.39)
46-64 years	2.35 (2.02-2.72)	2.67 (2.16-3.27)	1.14 (0.88-1.47)
65+ years	10.20 (8.80-11.76)	9.56 (7.65-11.81)	0.94 (0.72-1.22)

IR = Incidence Rate; CI = Confidence Interval; IRR = Incidence Rate Ratios

Supplementary Table 4. Incidence rate ratios (IRRs) for the burden of hospitalised pneumonia in adults at different time lag periods following the introduction of childhood PCV13 immunization

	**No lag** **IRR (95% CI)**	**3-month lag** **IRR (95% CI)**	**6-month lag** **IRR (95% CI)**	**12-month lag** **IRR (95% CI)**
**All-cause pneumonia**				
18-25 years	1.30 (0.94-1.79)	1.29 (0.94-1.78)	1.29 (0.93-1.78)	1.10 (0.79-1.51)
26-45 years	1.01 (0.79-1.28)	1.16 (0.91-1.48)	1.10 (0.87-1.41)	1.17 (0.92-1.48)
46-64 years	0.97 (0.76-1.25)	1.01 (0.78-1.29)	0.99 (0.77-1.28)	0.96 (0.75-1.23)
65+ years	1.03 (0.79-1.34)	1.08 (0.83-1.40)	0.99 (0.76-1.29)	0.86 (0.66-1.12)
**Severe pneumonia**				
18-25 years[1]	--	--	--	--
26-45 years	1.22 (0.65-2.29)	1.24 (0.66-2.34)	1.28 (0.68-2.42)	1.61 (0.86-3.02)
46-64 years	1.37 (0.82-2.27)	1.25 (0.75-2.07)	1.08 (0.65-1.80)	0.99 (0.60-1.64)
65+ years	0.97 (0.58-1.64)	0.97 (0.57-1.65)	0.98 (0.58-1.66)	1.10 (0.65-1.85)

[1]Not calculated due to small sample size; IRR = Incidence Rate Ratio; CI = Confidence Interval

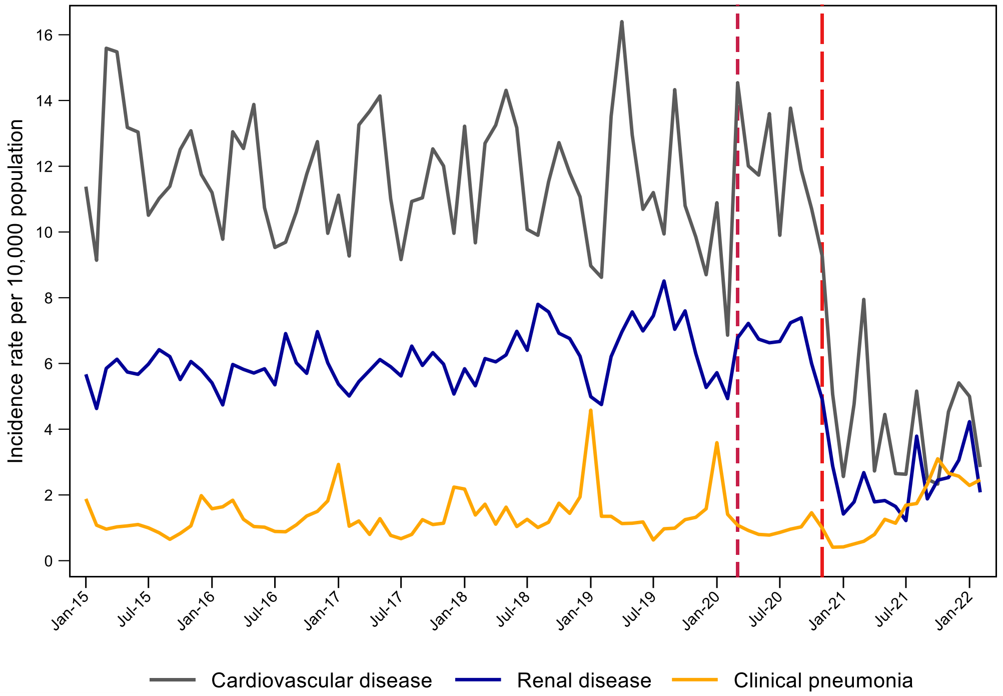

Supplementary Figure 1. Monthly incidence of adult (≥18 years) hospitalisation per 10,000 population, by hospital admission diagnosis. The vertical lines represent two key time points. The first dashed line represents the month WHO declared COVID-19 a global pandemic (March 2020).The second dashed line represents the month of Mongolia's first case of locally transmitted COVID-19 infection (November 2020).

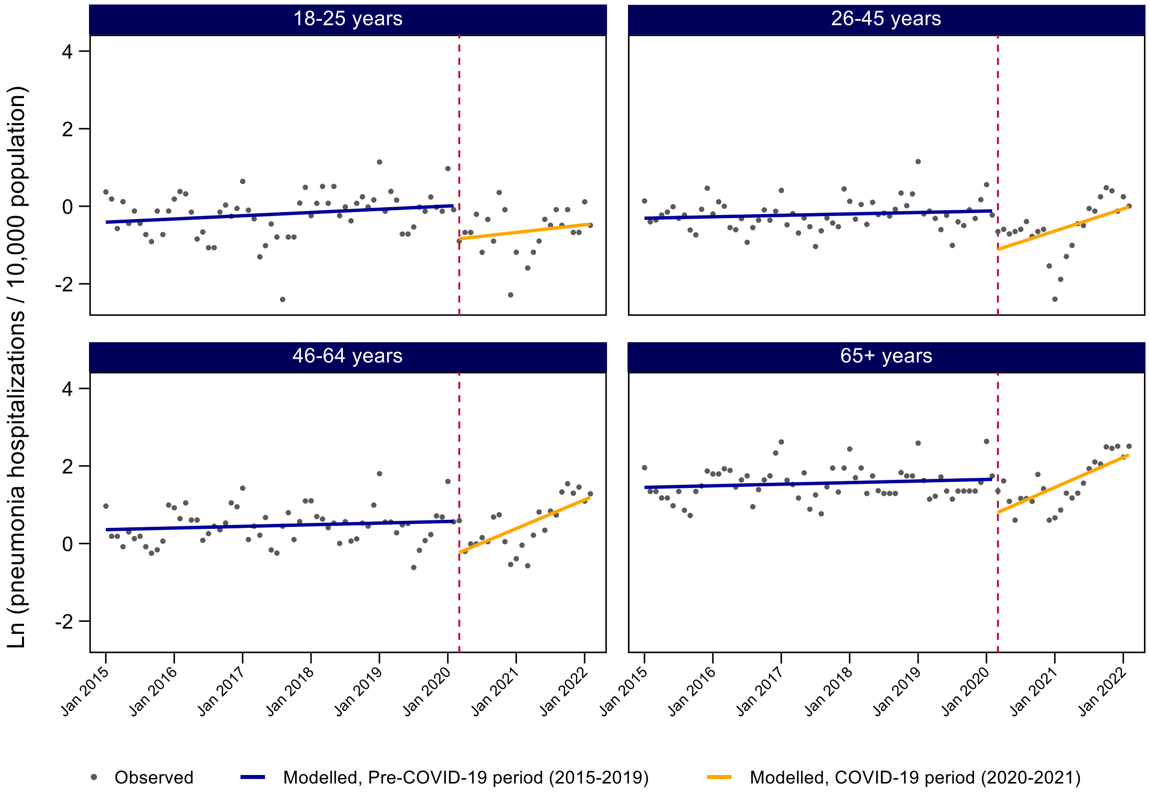

Supplementary Figure 2. Population-based incidence of adults (≥18 years) hospitalised with all-cause pneumonia for pre-COVID-19 and COVID-19 pandemic periods, per 10,000 population. The natural logarithm of the observed monthly hospitalised pneumonia incidence is plotted against the estimated hospitalised pneumonia incidence for pre-COVID-19 and COVID-19 periods. The red, dashed line represents the month WHO declared COVID-19 a global pandemic (March 2020).

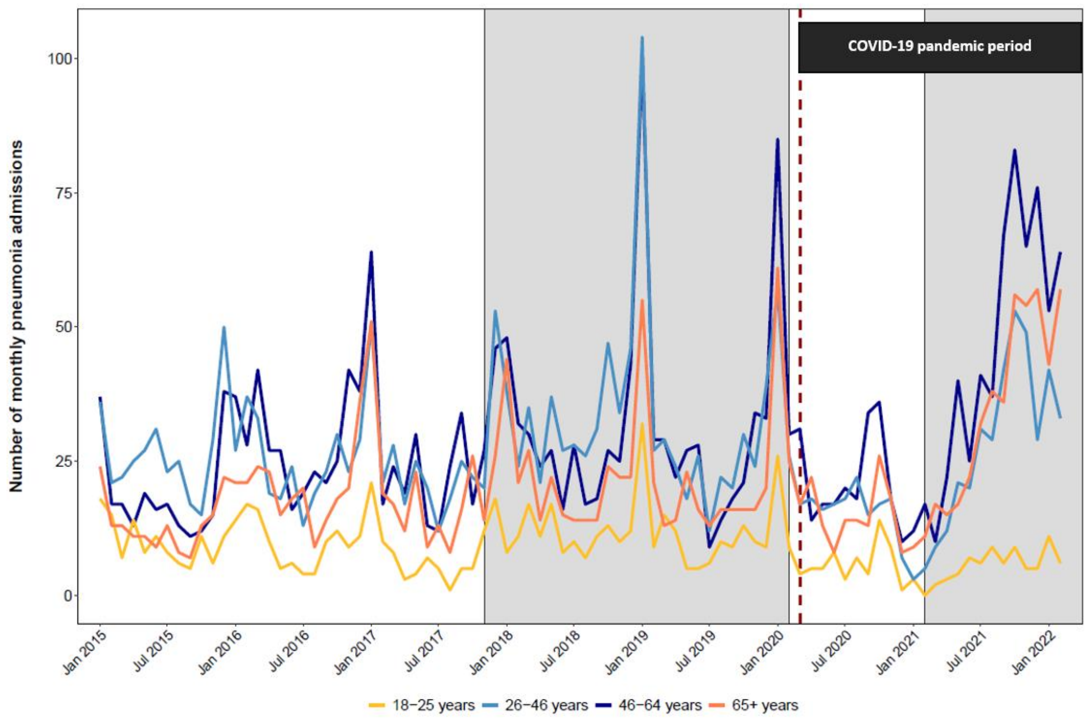

Supplementary Figure 3a. Results of breakpoint analysis on the number of all-cause pneumonia admissions across four district hospitals, by age group, January 2015—February 2022. The red dashed line indicates the month WHO declared COVID-19 a global pandemic (March 2020). White and grey areas represent segments with significant increases or decreases in pneumonia hospitalisations.

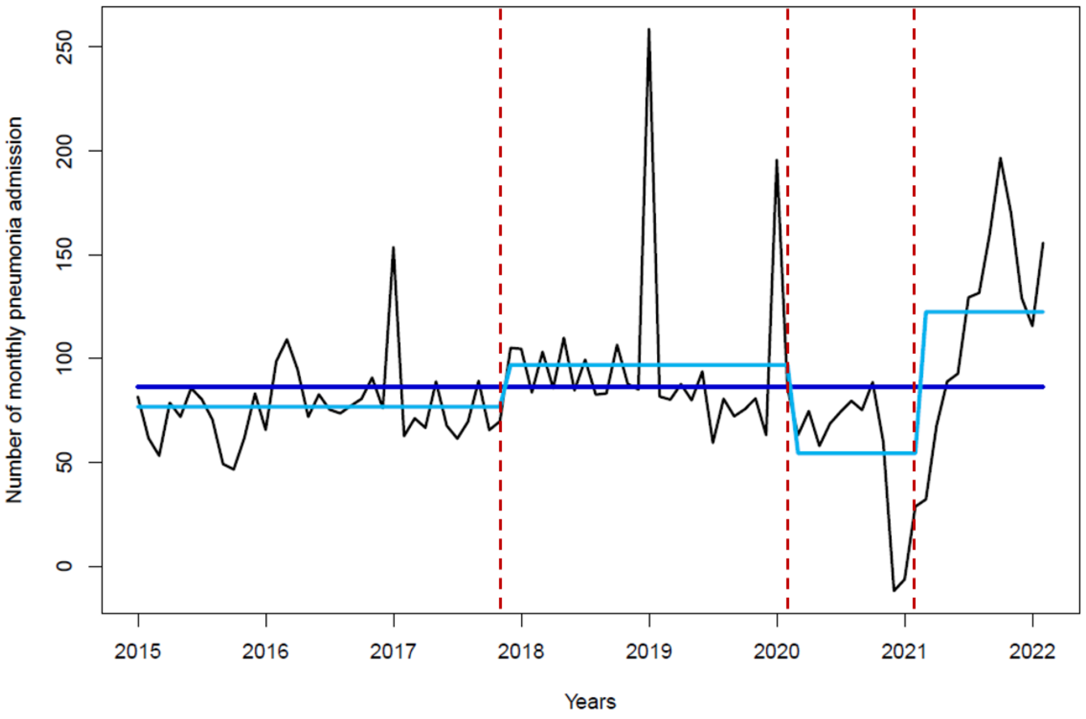

Supplementary Figure 3b. Trends in monthly adult all-cause pneumonia hospitalisation case counts and identified structural changes in time series, January 2015—February 2022. The black line shows monthly counts of all-cause pneumonia hospitalisations for adults ≥18 years. The red dashed lines indicate structural changes in the mean number of monthly pneumonia hospitalisations. The dark blue line shows the fitted mean value of the null model assuming no temporal changes in pneumonia hospitalisations. The light blue line represents significant increases or decreases in the estimated fitted mean values after the identified breakpoint.

References

1. Erkhembayar R, Dickinson E, Badarch D et al. Early policy actions and emergency response to the COVID-19 pandemic in Mongolia: experiences and challenges. *The Lancet Global Health* 2020; **8**(9): e1234-41.

2. Gombodorj G, Pető K. What Type of Households in Mongolia Are Most Hit by COVID-19? *Sustainability* 2022; **14**(6): 3557.

3. World Health Organization. Mongolia. Coronavirus Disease 2019 (COVID-19) Situation Report #94. 2022; Accessed 9th Jan 2023: https: //www.who.int/mongolia/internal-publications-detail/covid-19-situation-report-for-mongolia-94

4. Hale T, Angrist N, Goldszmidt R, et al. A global panel database of pandemic policies (Oxford COVID-19 Government Response Tracker). *Nature Human Behaviour* 2021; **5**(4): 529-38.

5. Ganzon JG, Xu L, Shehata DJ, et al. A perspective on impeding the COVID-19 pandemic: Lessons from Mongolia's comprehensive countermeasure. *Health Science Reports* 2023; **6**(1).

6. von Mollendorf C, Ulziibayar M, Gessner BD, et al. Evaluation of the impact of childhood 13-valent pneumococcal conjugate vaccine introduction on adult pneumonia in Ulaanbaatar, Mongolia: study protocol for an observational study. *BMC Public Health* 2021; **21**(1): 1731.

7. Zeileis A, Leisch F, Hornik K, Kleiber C. strucchange: An R package for testing for structural change in linear regression models. *Journal of Statistical Software* 2002; **7**: 1-38.

8. Cleveland RB, Cleveland WS, McRae JE, Terpenning I. STL: A seasonal-trend decomposition. *Journal of Official Statistics* 1990; **6**(1): 3-73.

9. Hyndman RJ, Khandakar Y. Automatic time series forecasting: the forecast package for R. *Journal of Statistical Software* 2008; **27**: 1-22.

10. Zeileis A, Kleiber C, Krämer W, Hornik K. Testing and dating of structural changes in practice. *Computational Statistics & Data Analysis* 2003; **44**(1-2): 109-23.
